# The importance of pre-operative neuroanatomical study in the surgical treatment of trigeminal neuralgia associated with multiple sclerosis

**DOI:** 10.3389/fnana.2023.1276977

**Published:** 2023-10-31

**Authors:** Nicola Montano, Alessandro Rapisarda, Quintino Giorgio D'Alessandris, Manuela D'Ercole, Alessandro Izzo

**Affiliations:** Neurosurgery Section, Department of Neuroscience, Fondazione Policlinico Universitario A. Gemelli IRCCS, Università Cattolica del Sacro Cuore, Rome, Italy

**Keywords:** trigeminal neuralgia, neurovascular conflict, multiple sclerosis, demyelination, neuroanatomy, microvascular decompression

## Introduction

Multiple sclerosis (MS) is a chronic, demyelinating disease of the central nervous system (CNS) occurring typically in younger patients, usually between 20 and 40 years of age. There are different clinical manifestations associated with MS, including visual impairment, sensory impairment, spasticity, urinary dysfunction, and pain. With advancing disease, the treatment of pain in MS patients becomes, along with MS-associated disability, the most problematic issue because there are different types of pain, such as thalamic or cortical deafferentation pain, extremity pain, and trigeminal neuralgia (TN) associated with MS, that can dramatically worsen the quality of life of these patients (Solaro et al., 2013; Di Stefano et al., 2019).

TN is the most frequent type of facial pain. Its incidence ranges from 12.6 to 27 per 100,000 per year (Montano et al., 2015), and from an etiological point of view, it can be divided into three subtypes. The classic subtype is usually related to the evidence of a neurovascular conflict at the trigeminal root entry zone due to the presence of an abnormal loop of a vascular structure, usually the superior cerebellar artery, causing nerve compression/distortion, atrophy, and demyelination (Baroni et al., 2023). The symptomatic TN type is secondary to the presence of different pathologies such as tumors of the cerebellopontine angle, arteriovenous malformations, otolaryngology diseases, and more frequently, MS. In the idiopathic form, no apparent cause is identifiable. In MS patients, TN is the most frequent form of neuropathic pain, with a prevalence ranging from 1.9% to 6.3% (Montano et al., 2015).

Many different factors can play a role in TN pathogenesis. In classical TN, demyelination in the region of nerve compression, ectopic generation of spontaneous nerve impulses, ephaptic conduction to adjacent fibers, and convergence of nociceptive afferents onto common central neurons are considered key mechanisms in the pathogenesis of pain. In MS-related TN, increased activity of T cells might lead to inflammatory activity in the plaques, triggering ephaptic nerve conduction and pain development (Zakrzewska et al., 2018).

The treatment of TN in MS patients can be challenging due to the fact that the antiepileptic drugs usually used for pain treatment, such as carbamazepine, oxcarbazepine, pregabalin, and lamotrigine, can cause different side effects and worsen the neurological symptoms of MS. Thus, surgical management of TN should be considered for MS patients who have uncontrolled pain despite antiepileptic drugs or show unacceptable side effects from drug assumptions (Bendtsen et al., 2019).

## Surgical options

There are different surgical options for the treatment of TN that can be divided into etiologic and palliative destructive procedures (Rapisarda et al., 2023). Microvascular decompression (MVD) is the only available etiological technique with the aim of removing the neurovascular conflict caused by the abnormal vessel loop on the trigeminal nerve root entry zone. Classically, MS patients have been excluded from this treatment due to the fact that the TN pathogenesis in these patients has been considered to be related to MS-associated demyelination. For these patients, the following palliative treatments have been advised: percutaneous destructive procedures involving a transforamen ovale approach to the retrogasserian portion of the trigeminal nerve; and gamma knife radiosurgery (GKRS) aiming at damaging the trigeminal nerve root, impairing the nerve fiber transmission, resulting in pain relief.

## Discussion

Recently, the classic paradigm of MS demyelination as the exclusive cause of trigeminal pain onset in these patients has been questioned. It has been reported that in many patients with MS, a pontine demyelinating plaque and a neurovascular compression on the trigeminal nerve can co-exist (Truini et al., 2016). Moreover, there are MS patients in whom, despite the presence of trigeminal pain, no demyelinating plaque is evident on the trigeminal anatomical pathway (Montano et al., 2019). In our opinion, these data should lead us to reconsider using MVD to treat TN in patients with MS. In fact, although efficient with immediate pain relief in 80% to 90% of cases and a pain-free survival ranging from 2 to 3 years (Montano et al., 2012, 2019; Zakrzewska et al., 2018), percutaneous techniques carry a high risk of trigeminal sensory loss with trigeminal dysesthesias and corneal numbness leading to keratitis. Furthermore, in some cases, mastication weakness can develop after the operation (Tatli et al., 2008). Moreover, a systematic review of GKRS use in patients with MS demonstrated lower results compared with the other procedures (Montano et al., 2013). On the other hand, although more invasive, MVD is associated with a lower risk of sensory disturbances, and a recent systematic review evidenced acute pain relief in 71.42% of MS-related TN cases (Montano et al., 2020).

Some recent pieces of evidence suggest that the presence of a demyelinating plaque related to TN is associated with a better outcome after percutaneous balloon compression (Montano et al., 2019) and that patients who do not have a plaque near the trigeminal pathway may experience prolonged clinical benefit from MVD (Paulo et al., 2020). A systematic review seems to confirm these data (Montano et al., 2020). Moreover, a recent study suggests that MVD can be offered in patients with TN secondary to MS when there is a neurovascular conflict (Worm et al., 2023).

**Figure 1:**
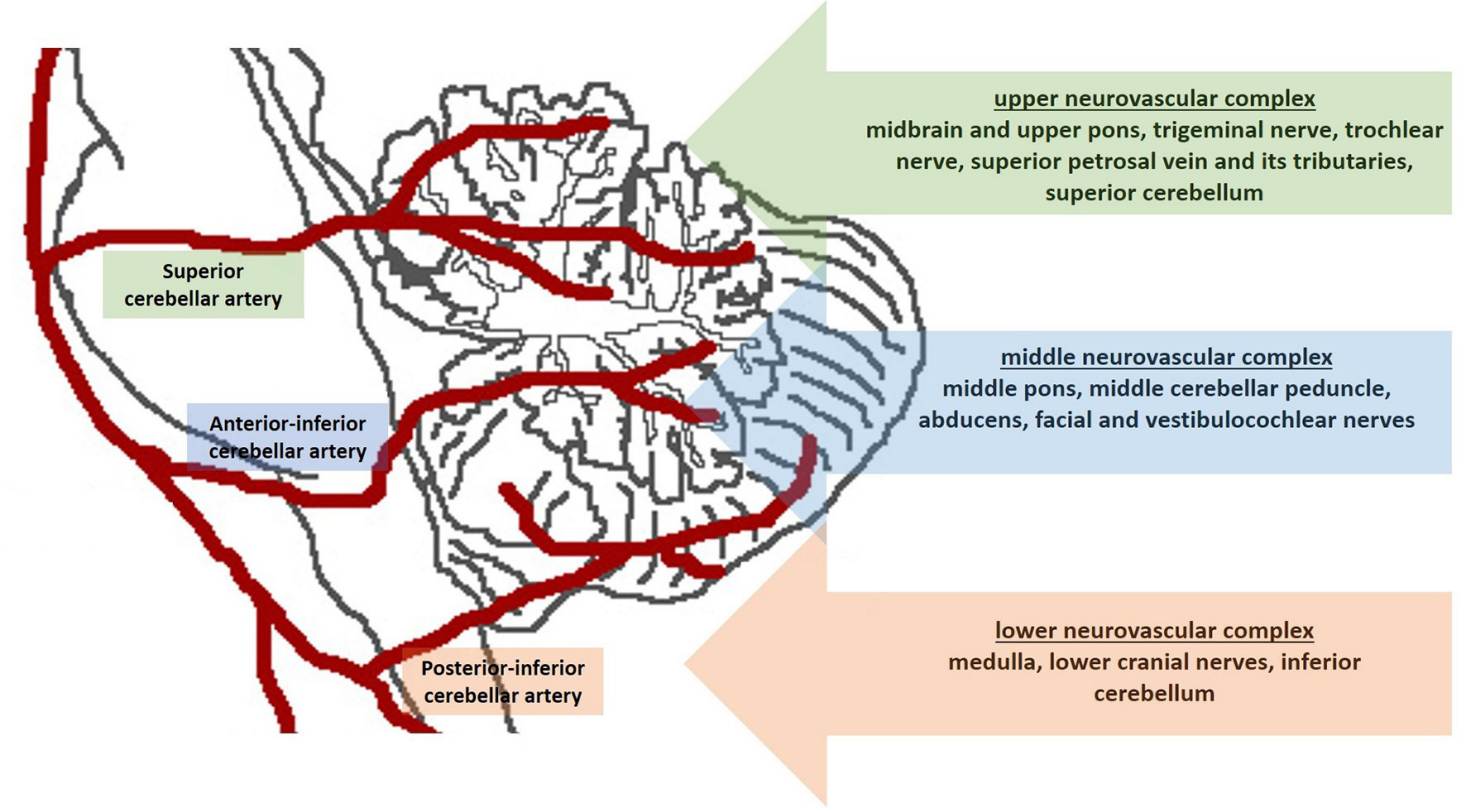
Schematic figure shows the “anatomical rule of three”. The cerebellopontine angle is divided into three compartments according to the cerebellar arteries.

In our practice, we consider the pre-operative neuroanatomical study of the trigeminal region mandatory for these patients. Rhoton proposed the “anatomical rule of three” (Figure 1) and divided the cerebellopontine angle into three compartments according to the cerebellar arteries arising from the vertebrobasilar system (Rhoton, 2000). Briefly, the “upper neurovascular complex” follows the superior cerebellar artery and includes the midbrain and upper pons, trigeminal nerve, trochlear nerve, superior petrosal vein and its tributaries, and superior cerebellum. The “middle neurovascular complex” follows the anterior inferior cerebellar artery and includes the middle pons, middle cerebellar peduncle, abducens, and facial and vestibulocochlear nerves. The “lower neurovascular complex” follows the posterior inferior cerebellar artery and includes the medulla, lower cranial nerves, and inferior cerebellum (Rhoton, 2000).

To carefully study the neuroanatomy of this compartment, in the pre-operative workup of these patients, we always perform magnetic resonance imaging with three-dimensional fast imaging employing steady-state acquisition (FIESTA) sequences. In a recent report, these thin-cut sequences, offering a good contrast between the CSF (hyperintense), cranial nerves (isointense), and cerebellar arteries (hypointense due to the flow voids), have shown good reliability in predicting the neurovascular conflict in patients with TN (Hu et al., 2022).

**Figure 2:**
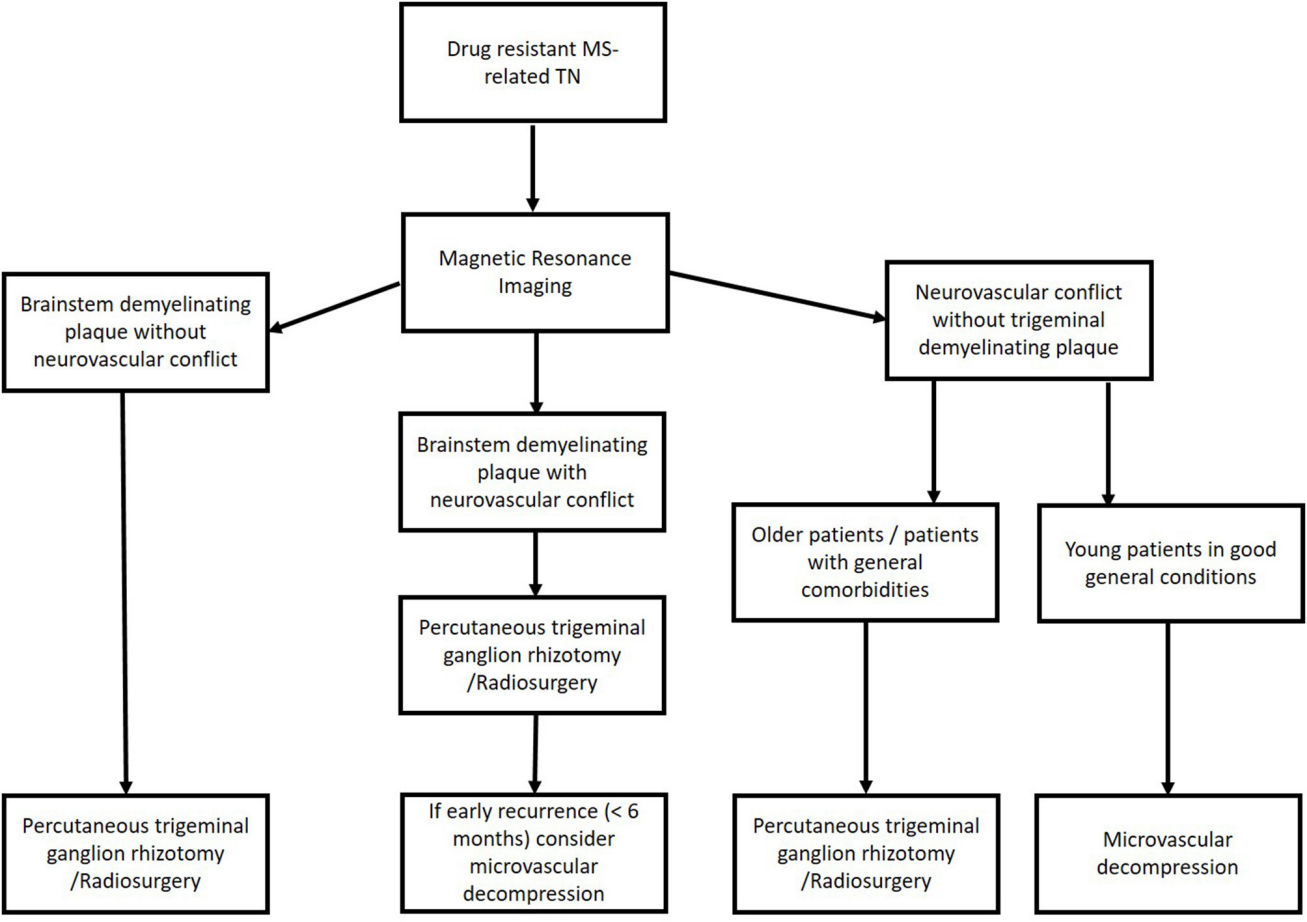
Flowchart to treat patients with multiple sclerosis-related trigeminal neuralgia.

Based on these data, we use the flowchart reported in Figure 2 to treat patients with MS-related TN (Figure 2). Briefly, in young patients with evidence of neurovascular conflict and the lack of a demyelination plaque, we consider MVD to be the first choice to treat MS-related drug-resistant TN. If we have an MS patient with both neurovascular conflict and a demyelinating plaque, we consider a palliative technique as the first choice and MVD in cases of an early recurrence of pain. In cases without neurovascular conflict, a palliative procedure is a reasonable option.

We systematically applied this flowchart in January 2021. We treated 30 MS-related TN patients. Of them, 22 were submitted to percutaneous balloon compression, and eight were treated with MVD. The clinical and outcome data of patients are reported in Table 1. Briefly, we had a statistically significant improvement in pain measured by the visual analog scale (VAS) and the Barrow Neurological Institute (BNI) scale (*p* < 0.0001 and *p* < 0.0001, respectively). Regarding the complications, one patient developed transient mild facial nerve palsy after MVD and completely recovered after 2 months, while another patient developed transient diplopia after the percutaneous treatment and totally recovered after 4 weeks. At the latest follow-up (FU), we observed no recurrence in the MVD group and only three recurrences (13.63%) in the percutaneous balloon compression group.

**Table 1:** Clinical and outcome data of MS related TN patients submitted to surgical treatment.

Age (years)	56.72 ± 10.03
Sex (M/F)	14/16
MS duration before operation (years)	16.75 ± 7.53
TN duration before operation (years)	8.33 ± 5.57
Operation (MVD/percutaneous)	8/22
VAS before operation	9.61 ± 0.66
VAS at FU	0.88 ± 2.08
BNI before operation	4.42 ± 0.59
BNI at FU	1.27 ± 0.66
FU (months)	12.88 ± 8.55

TN, trigeminal neuralgia; MS, multiple sclerosis; MVD, microvascular decompression; VAS, visual analogue scale; FU, follow-up; BNI: Barrow Neurological Institute.

In conclusion, the surgical treatment of drug-resistant TN in MS patients is a challenge for neurosurgeons. The full knowledge of the pre-operative neuroanatomy of each patient can help the neurosurgeon choose the correct surgical procedure, improving the quality of life of these patients.

## Author contributions

NM: Conceptualization, Writing—original draft, Writing—review & editing. AR: Investigation, Writing—review & editing. QD'A: Formal analysis, Writing—review & editing. MD'E: Investigation, Methodology, Writing—review & editing. AI: Validation, Writing—review & editing.
